# Brain-computer interface-based robotic end effector system for wrist and hand rehabilitation: results of a three-armed randomized controlled trial for chronic stroke

**DOI:** 10.3389/fneng.2014.00030

**Published:** 2014-07-29

**Authors:** Kai Keng Ang, Cuntai Guan, Kok Soon Phua, Chuanchu Wang, Longjiang Zhou, Ka Yin Tang, Gopal J. Ephraim Joseph, Christopher Wee Keong Kuah, Karen Sui Geok Chua

**Affiliations:** ^1^Institute for Infocomm Research, Agency for Science, Technology and Research (A*STAR)Singapore; ^2^Department of Rehabilitation Medicine, Tan Tock Seng HospitalSingapore

**Keywords:** electroencephalography, motor imagery, brain-computer interface, stroke rehabilitation, robotic

## Abstract

The objective of this study was to investigate the efficacy of an Electroencephalography (EEG)-based Motor Imagery (MI) Brain-Computer Interface (BCI) coupled with a Haptic Knob (HK) robot for arm rehabilitation in stroke patients. In this three-arm, single-blind, randomized controlled trial; 21 chronic hemiplegic stroke patients (Fugl-Meyer Motor Assessment (FMMA) score 10–50), recruited after pre-screening for MI BCI ability, were randomly allocated to BCI-HK, HK or Standard Arm Therapy (SAT) groups. All groups received 18 sessions of intervention over 6 weeks, 3 sessions per week, 90 min per session. The BCI-HK group received 1 h of BCI coupled with HK intervention, and the HK group received 1 h of HK intervention per session. Both BCI-HK and HK groups received 120 trials of robot-assisted hand grasping and knob manipulation followed by 30 min of therapist-assisted arm mobilization. The SAT group received 1.5 h of therapist-assisted arm mobilization and forearm pronation-supination movements incorporating wrist control and grasp-release functions. In all, 14 males, 7 females, mean age 54.2 years, mean stroke duration 385.1 days, with baseline FMMA score 27.0 were recruited. The primary outcome measure was upper extremity FMMA scores measured mid-intervention at week 3, end-intervention at week 6, and follow-up at weeks 12 and 24. Seven, 8 and 7 subjects underwent BCI-HK, HK and SAT interventions respectively. FMMA score improved in all groups, but no intergroup differences were found at any time points. Significantly larger motor gains were observed in the BCI-HK group compared to the SAT group at weeks 3, 12, and 24, but motor gains in the HK group did not differ from the SAT group at any time point. In conclusion, BCI-HK is effective, safe, and may have the potential for enhancing motor recovery in chronic stroke when combined with therapist-assisted arm mobilization.

## Introduction

Stroke is the third leading cause of severe disabilities worldwide (Hankey, 2013). Despite multimodality rehabilitation efforts, 40% of stroke survivors live with various disabilities. Of these, the lack of functional arm, wrist, or hand recovery contributed to significant losses in independence vocation and quality of life. Task specific technique such as constrained-induced movement therapy (CIMT) is highly effective in reducing learned non-use and improving arm and hand function with enduring gains in chronic stroke. However, only ~20 to 25% of stroke patients meet minimum criteria for CIMT (Fritz et al., 2005). Since physical practice (PP) of the stroke-impaired extremity is often difficult or not possible using CIMT; motor imagery (MI), the mental practice of movements without physical execution, represents an alternate rehabilitation approach (Sharma et al., 2009). Although MI in chronic stroke is promising, integrating MI in rehabilitation had yielded inconclusive clinical outcome (Braun et al., 2006; Ietswaart et al., 2011; Malouin et al., 2013).

One of the key issues for integrating MI in rehabilitation is that while PP is observable, MI is a concealed mental process. Nevertheless, brain-computer interfaces (BCIs) (Wolpaw et al., 2002) that acquire, analyze and translate brain signals into control commands of output devices (Shih et al., 2012) can be used to detect event-related desynchronization or synchronization (ERD/ERS) (Pfurtscheller and Lopes Da Silva, 1999) when MI is performed. In this way, stroke patients who suffer from severe limb weakness but who are still able to imagine movements of the paretic hand can receive BCI contingent feedback upon detection of MI-related brain signals (Birbaumer et al., 2008; Buch et al., 2008; Ramos-Murguialday et al., 2013). By re-establishing contingency between cortical activity related to MI and feedback, BCI might strengthen the sensorimotor loop and foster neuroplasticity that facilitates motor recovery (Dobkin, 2007; Dimyan and Cohen, 2011). A recent clinical study had shown that Electroencephalography (EEG)-based MI-BCI can be used to detect cortical activity related to MI in a majority of stroke patients (Ang et al., 2011). Hence the use of EEG-based MI-BCI presents a prospective approach for detecting MI for stroke rehabilitation.

There were many studies that reported the use of BCI for stroke rehabilitation (Ang and Guan, 2013). Recent trials that reported clinical efficacy included: Mihara et al. (2013) reported a randomized control trial (RCT) performed on 10 stroke patients who received near-infrared spectroscopy (NIRS)-based MI-BCI with visual feedback vs. 10 stroke patients who received NIRS-based MI-BCI with irrelevant feedback. The results showed that the patients who received MI-BCI visual feedback attained significantly greater motor improvements measured using Fugl-Meyer motor assessment (FMMA) (Fugl-Meyer et al., 1975) compared to the sham group. The FMMA is a well-designed, feasible, and efficient clinical examination method that has been widely used in the stroke population for measuring sensorimotor stroke recovery (Gladstone et al., 2002). The motor score ranges from 0 for hemiplegia to a maximum of 100 points for normal motor performance, divided into 66 points for the upper extremity and 34 points for the lower extremity. Ramos-Murguialday et al. (2013) reported a RCT on 16 chronic stroke patients who received MI-BCI with hand and arm orthoses feedback vs. 14 chronic stroke patients who received random orthoses feedback not linked to BCI. Both groups received physiotherapy, and the results showed that the patients who received BCI orthoses feedback attained significantly greater motor improvement in FMMA score. Recently, Ang et al. (2014) reported a RCT on 11 chronic stroke patients who received MI-BCI with MIT MANUS shoulder-elbow robotic feedback vs. 15 chronic stroke patients who received intense movement exercises using the MIT MANUS robot. The results showed the patients who received MI-BCI intervention attained an average of FMMA gains of 4.5, and the patients who received intense robot-assisted movement therapy attained an average of FMMA gains of 6.3. However, no significant differences between the two groups were found.

In a systematic review, Nilsen et al. (2010) attested that MI added to PP was an effective intervention for stroke. However, existing RCTs have demonstrated motor improvements in chronic stroke patients who received MI-BCI intervention, but there is still scanty clinical efficacy to indicate the benefits of performing MI compared to PP or standard arm therapy (SAT) in stroke rehabilitation (Ietswaart et al., 2011). Hence we sought to investigate the clinical benefits of concomitant MI, PP interventions for stroke rehabilitation by integrating MI and PP using an EEG-based MI-BCI coupled with a haptic knob (HK) robot (Lambercy et al., 2007, 2011). We then investigated the hypothesis that this integration could facilitate the beneficial effects of therapist-assisted arm mobilization for stroke patients compared to robot-assisted PP and SAT in current rehabilitation program.

## Materials and methods

### Ethics statement

Ethics Committee approval was obtained from the Institution's Domain Specific Review Board, National Healthcare Group, Singapore. The trial was registered in ClinicalTrials.gov (NCT01287975). Informed consent was obtained prior to study enrollment.

### Study design

**Figure 1:**
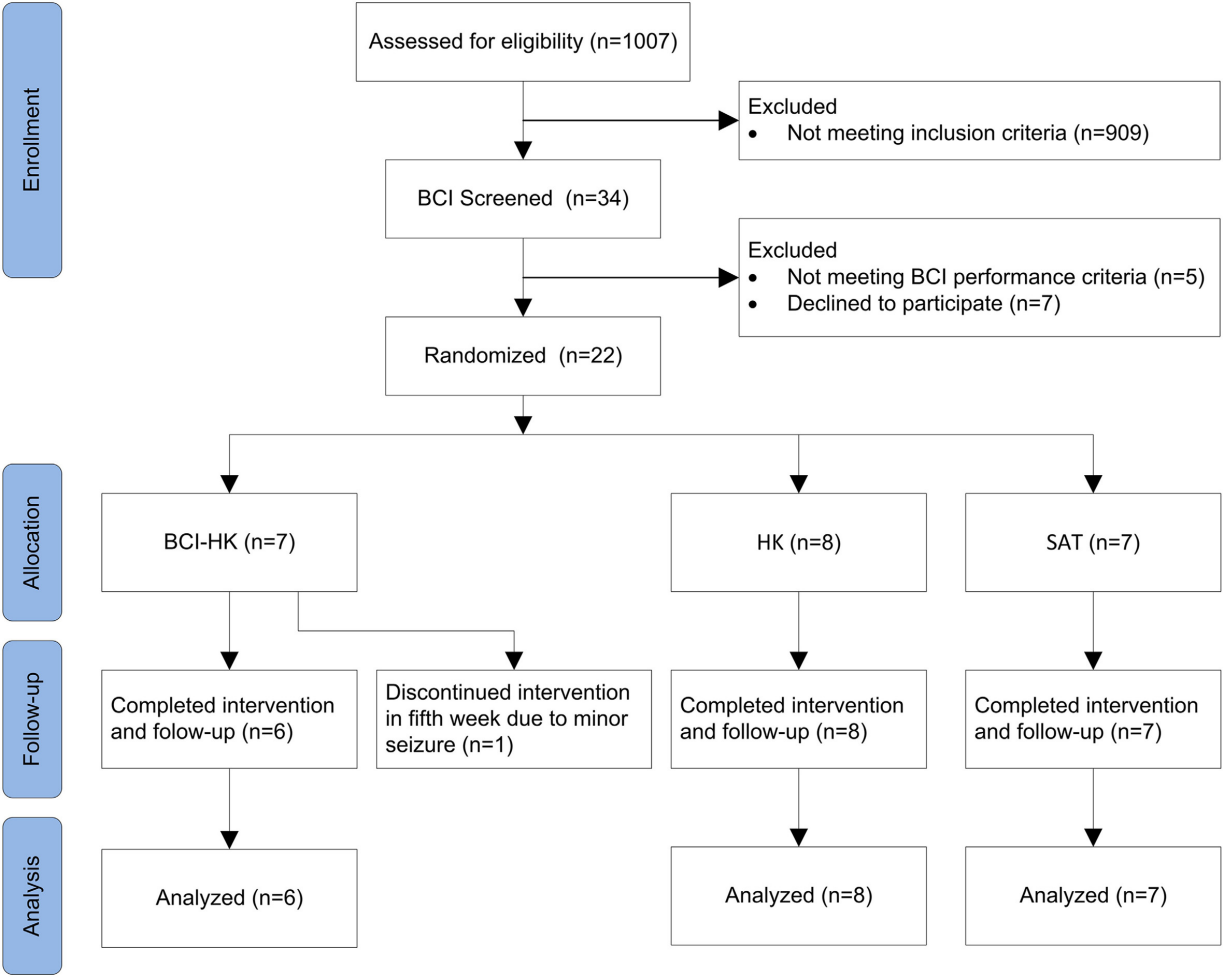
**CONSORT Diagram: a flow from recruitment through follow-up and analysis**.

The randomized controlled trial was conducted over ~2.5 year period from 1 January 2011 to 31 June 2013 at an outpatient rehabilitation facility, involving subjects who had completed inpatient rehabilitation at the Tan Tock Seng Hospital, Singapore. Figure 1 shows a flow chart of the trial (refer Supplementary Material for CONSORT checklist).

Inclusion criteria included first-ever clinical stroke confirmed on neuroimaging, ages 21–80 years of age, duration >4 months post stroke, moderate to severe impairment of upper extremity function assessed by FMMA (Fugl-Meyer et al., 1975) score 10–50; motor power assessed by Medical Research Council (MRC) (Compston, 2010) grades >2/5 in shoulder abductors, and >2/5 in the elbow flexors, and 1–3 in wrist dorsiflexors and finger flexors and ability to understand simple instructions.

Subjects were excluded if they had medical instability such as unresolved sepsis; postural hypotension; end stage renal failure terminal illness; severe aphasia, inattention; hemi spatial neglect; severe visual impairment; epilepsy; severe depression; psychiatric disorder; recurrent stroke; skull defects compromising EEG cap fit; severe spasticity assessed [modified Ashworth scale (MAS) (Bohannon and Smith, 1987) >2 in any shoulder, elbow or wrist/finger muscles]; pain assessed by visual analog scale (VAS) (Price et al., 1999) >4/10; fixed joint contractures; skin conditions such as infections or eczema which could be worsened by robotic exoskeletal or EEG cap contact.

### EEG data acquisition

In this study, EEG data from 27 channels were collected using the Nuamps EEG acquisition hardware^1^ with unipolar Ag/AgCl electrodes channels, digitally sampled at 250 Hz with a resolution of 22 bits for voltage ranges of ±130 mV. EEG recordings from all channels were bandpass filtered from 0.05 to 40 Hz by the acquisition hardware.

### Haptic knob robot

The haptic knob (HK) robot is a two-degree-of-freedom robotic hand interface for hand grasping and knob manipulation PP (Lambercy et al., 2007, 2011). The hand interface was designed using two parallelogram structures that supported an exchangeable handle in order to adapt to various hand sizes, finger orientations, and subjects with right or left stroke-impaired hand. The HK robot-assisted hand grasping PP involved finger flexion and extension exercises performed using the linear degree-of-freedom (DOF) of the HK, while the rotational DOF was held in a static position. The HK robot-assisted knob manipulation PP involved wrist pronation or supination, and hand coordination exercises performed using the rotational DOF of the HK, while the linear DOF was held in a static position.

During training with the HK, subjects were seated comfortably in a padded, height adjustable chair with 2-point chest strapping without arm rests to reduce compensatory trunk movements. For each subject, the stroke-impaired forearm was placed on a padded support and the subject was instructed to grasp the end effector of the HK. The height of the chair was adjusted until a comfortable level, the subject's shoulder abducted at about 40° and the elbow flexed at about 90°. The digits of the subject stroke-impaired hand were then strapped to the HK's end effector with Velcro bands to prevent them from slipping.

**Figure 2:**
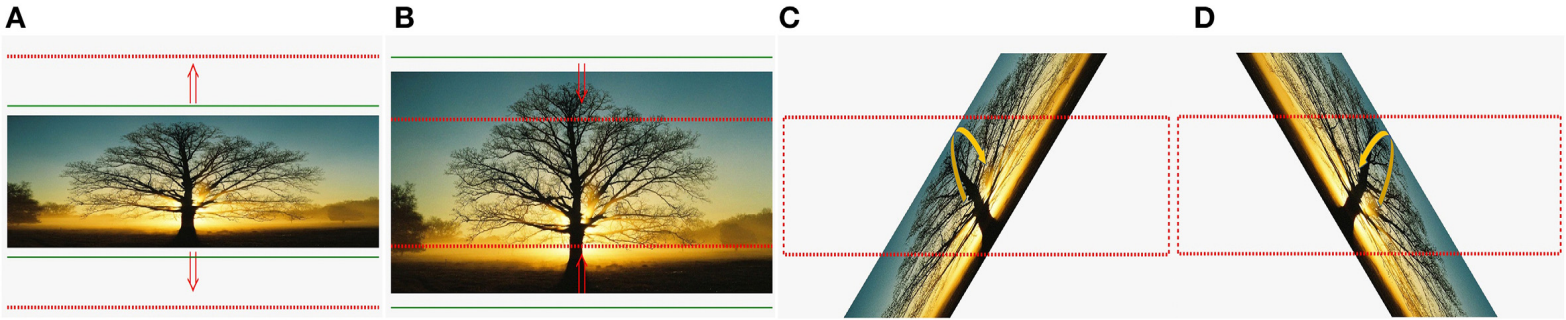
**Cues used in BCI-HK and HK interventions**. **(A)** Hand opening; **(B)** hand closing; **(C)** wrist pronation; and **(D)** wrist supination.

Instructions and feedbacks were provided on a computer screen for the progress of the HK robot-assisted PP in a form of a picture manipulation task using a solid frame to represent the current position, and a dotted frame to represent the target position. For the HK robot-assisted hand grasping PP, an outward-pointing arrow was shown to instruct the subject to perform hand opening (Figure 2A). Once the target outer limit was reached, an inward-pointing arrow was shown to instruct the subject to perform hand closing (Figure 2B). This open-and-close action formed a single trial. Subsequently, a different picture was used for the next trial. For the HK robot-assisted knob manipulation PP, a right-curved arrow was shown to instruct the subject to perform a clockwise wrist rotation (Figure 2C). Once the target limit is reached, a left-curved arrow was shown to instruct the subject to perform counter-clockwise wrist rotation (Figure 2D). This wrist pronation-and- supination action formed a single trial. Similarly, a different picture was used for the next trial. For both the hand grasping and knob manipulation PP, HK robot-assisted movement was initiated if no movement from the subject was detected after an interval of 2 s.

### EEG-based MI-BCI screening

A study on 99 healthy subjects had shown that ~7% of the subjects achieved below 60% classification accuracies (Guger et al., 2003). Subsequently, a study on 54 stroke patients had shown that ~13% of the patients achieved classification accuracies below chance level (Ang et al., 2011). Hence there is a small minority of subjects who cannot operate EEG-based MI-BCI. Thus, in this study, eligible subjects were first screened for their ability to operate EEG-based MI-BCI.

**Figure 3:**
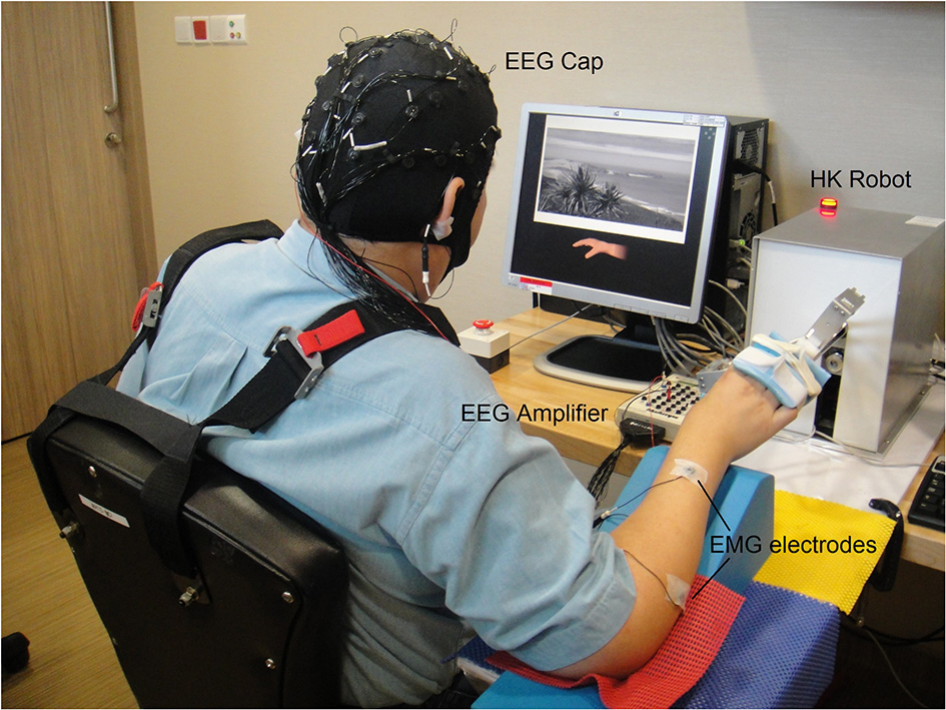
**Setup of BCI-HK and HK intervention for stroke rehabilitation at a local hospital**. The setup comprised Electroencephalography (EEG) cap, Electromyography (EMG) electrodes, EEG amplifier, and Haptic Knob (HK) robot.

**Figure 4:**
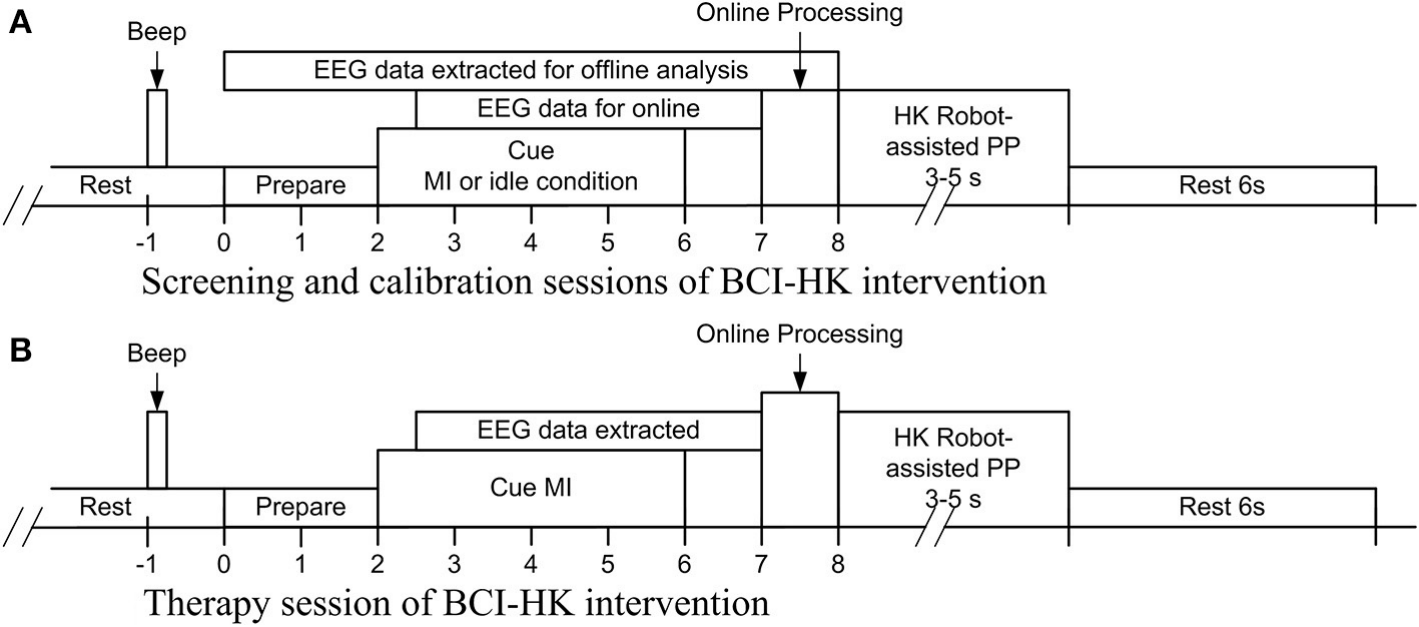
**Acquisition of EEG for BCI-HK intervention**. **(A)** Timing of performing kinesthetic MI of the stroke-impaired hand and idle condition for the calibration session; **(B)** timing of performing kinesthetic MI of the stroke-impaired coupled with HK robot-assisted PP for the rehabilitation session.

The screening session comprised 4 runs of EEG data collection. The first 2 runs collected EEG from subjects who performed kinesthetic MI (Stinear et al., 2006) of the stroke-impaired hand while strapped to the HK, and idle condition. The subjects were seated comfortably and instructed to imagine moving their stroke-impaired hand in an open-and-close action, and voluntary movements were restrained by static resistance from the HK robot. Subjects were also instructed to minimize voluntary head and body movements. Electromyography (EMG) were recorded from the stroke-impaired hand to check for attempted movements while performing motor imagery (Figure 3). In the subsequent 2 runs, the subjects were instructed to relax while passive movement (PM) of the stroke-impaired hand was performed using the HK robot for the hand grasping action. The entire screening session consisted of 4 runs of 80 trials each for a total of 320 trials, and an inter-run break of at least 2 min was provided. Each run comprised 40 trials of MI or PM, and 40 trials of idle condition. Figure 4A shows the timing for a single-trial from the screening section. Each trial lasted ~12 s and each run lasted ~8 min. The screening session lasted ~1 h inclusive of EEG setup time. The EEG from the first 2 runs were used to compute the 10×10-fold cross-validation accuracy of classifying MI of the stroke-impaired hand vs. the idle condition using the filter bank common spatial pattern (FBCSP) algorithm (Ang et al., 2012).

### Randomization and blinding

Subjects who passed BCI screening were randomly assigned to receive either one of 3 interventions:

1. BCI-HK which concomitantly comprised EEG-based MI-BCI coupled with HK robot-assisted PP therapy (60 min) followed by therapist-assisted arm mobilization (30 min).
2. HK which comprised HK robot-assisted PP therapy (60 min) followed by therapist-assisted arm mobilization (30 min).
3. SAT which comprised distal arm training of forearm pronation-supination movements incorporating wrist control and grasp-release of various objects (60 min) and overall therapist-assisted arm mobilization (30 min) conducted by a trained occupational therapist.

The randomization block size was 3 and the allocation sequence was 1:1:1 generated using STATA software version 10.2 (Stata Corp, College Station, TX, USA). Enrollment and assignment of participants was provided by KSGC. As subject blinding was not feasible, all outcome assessments for this study were performed by occupational therapist DXD who was blinded to allocation. There were no protocol deviations.

All groups received 18 sessions of supervised interventions for a total of 27 h over 6 weeks, 3 times per week, 90 min per session, by occupational therapist GJEJ and engineer KSP. This included 15 min for set up and breaks for short rests. Adverse events via questionnaire were monitored after each intervention session. Discontinuation criteria included new neurological or serious adverse events; increase in arm pain or spasticity of greater than 30% from baseline; or severe fatigue resulted from the interventions.

#### BCI-HK intervention

The BCI-HK intervention consisted of a calibration session and 18 therapy sessions of MI-BCI coupled with HK robot-assisted PP therapy. Figure 3 shows the setup for the BCI-HK intervention.

The calibration session comprised 4 runs of EEG data collection that was similar to the screening session whereby subjects performed MI in the first 2 runs and PM in the subsequent 2 runs. The EEG from the first 2 runs were used to compute a subject-specific calibration model using the FBCSP algorithm (Ang et al., 2012) to classify MI vs. the idle condition in the subsequent therapy sessions. The EEG data collected from performing PM were not analyzed in this study.

Each therapy session comprised 4 runs of 30 concomitant MI and PP trials each, for a total of 120 trials. An inter-run break of 3–5 min was provided after each run. Allowable pain-free ranges of motion for the hand grasping and knob manipulation PP were first individually pre-determined by GJEJ. This HK calibration involved calibrating six positions: closing, opening, and static position for hand grasping; clockwise, counter clockwise and static position for knob manipulation. For the first 2 runs, the subjects are instructed to perform kinesthetic MI of the stroke-impaired hand for the hand grasping action. Subjects were also instructed to minimize voluntary head and body movements. EMG from the stroke-impaired hand was checked to ensure that there was no attempted movement during MI. If MI-related brain signals was successfully detected by the FBCSP algorithm (Ang et al., 2012) using the subject-specific calibration model, then the HK robot-assisted hand grasping PP would be initiated. For the subsequent 2 runs, the subjects are instructed to perform kinesthetic MI of the stroke-impaired hand for the knob manipulation action. If MI was successfully detected, then the HK robot-assisted knob manipulation PP would be initiated. If MI was not detected in 2 consecutive trials, then the HK robot-assisted PP would be automatically initiated. Figure 4B shows the timing for a single-trial from the therapy session. Each trial lasted ~17 to 23 s and each run lasted ~12 min. Each therapy session lasted ~1.5 h inclusive of breaks and setup time.

#### HK intervention

The HK intervention comprised 18 therapy sessions of HK robot-assisted hand grasping, and knob manipulation PP. Figure 3 shows the setup for the HK intervention, which is the same as the BCI-HK intervention. EEG data was also collected for the HK intervention but was not analyzed in this report.

Each therapy session comprised 4 runs of 30 PP trials each for a total of 120 trials. An inter-run break of 3–5 min was provided after each run. Similar to the BCI-HK intervention, allowable pain-free ranges of motion were pre-determined, and HK calibration was performed by GJEJ prior to the start of the therapy session. For the first 2 runs, the subjects performed HK robot-assisted hand grasping PP. For the subsequent 2 runs, the subjects performed HK robot-assisted knob manipulation PP. If no movements from the subject were detected, the HK would initiate fully assisted PP after 2 s. Each trial lasted ~9 to 15 s and each run lasted ~8 min. Each therapy session lasted ~ 1 h inclusive of breaks and setup time.

#### SAT intervention

The SAT intervention comprised 18 therapist-assisted sessions. Each session comprised 60 min of repetitive task training (Langhorne et al., 2011) focusing on forearm pronation-supination movements incorporating wrist control and grasp-release of various objects.

#### Therapist-assisted arm mobilization

All 3 groups received 30 min of therapist-assisted arm mobilization following the principles of the professionally recognized Neuro-developmental Treatment Approach for stroke rehabilitation (Howle, 2002), which included tone management and facilitation toward normal arm movement patterns via various closed-chain functional reach activities.

### Sample size statistical analysis

The sample size was estimated with an assumption of a 4 point gains in total FMMA score for the BCI-HK and HK groups compared to the SAT group, and a standard deviation of 6.3 points based on the gains of the robot-assisted intervention in the previous study (Ang et al., 2014). The expected number in each group was found to be 20 subjects to achieve statistical power of 80%. Sample size calculation was performed in MATLAB.

### Statistical methods

Analysis of variance (ANOVA) was used to examine the demographic and baseline group differences. Analysis of covariance (ANCOVA) was used to examine the group differences at each measurement point between the three groups after adjusting for baseline differences. Two-sided *t*-tests were performed to analyze for significant difference at each measurement point from baseline in each group. One-sided *t*-tests were then performed to analyze if the BCI-HK and HK interventions were better than the SAT intervention. Data analysis was performed using MATLAB and the level of significance was set at 5%.

### Outcomes

The primary outcome was the total FMMA score (range, 0–66) for the stroke-impaired upper extremity. Outcomes were measured at 5 time points during the study: at baseline (week 0), at mid-intervention (week 3), at completion of intervention (week 6), 6 weeks follow-up (week 12), and 18 weeks follow-up (week 24). There were no protocol deviations.

## Results

### Patient enrollment

Thirty-four subjects were found eligible and subsequently screened for their ability to use EEG-based MI BCI. The EEG data collected from the screening session showed 5 subjects achieved accuracies that were lower than chance level (57.5%) and were thus excluded. The chance level performance was computed based on 95% confidence estimate of the accuracy using the inverse of binomial cumulative distribution. Seven subjects declined further participation in the trial. The remaining 22 subjects gave consent and were randomized into 3 intervention groups as follows: BCI-HK (7 subjects), HK (8 subjects) and SAT (7 subjects) respectively. Twenty-one subjects completed the study and follow-up with 1 drop out (4.6%) (Figure 1). The study terminated in June 2013 due to funding cessation, thus not all 60 intended subjects could be recruited.

Table 1 shows the demographic of the 21 subjects who completed the study by intervention. Altogether, there were 14 men and 7 women [mean age 54.2 years (30–79)], mean stroke duration, 385.1 days (191–651). BCI-HK group had more subcortical strokes, shorter time after the stroke, and higher FMMA score at week 0. SAT group had higher proportion of cerebral infarctions compared to hemorrhagic strokes. There were no significant baseline differences in all 3 groups in terms of stroke type [*F*_(2, 18)_ = 0.90, *p* = 0.42], stroke nature [*F*_(2, 18)_ = 0.53, *p* = 0.60], duration since stroke [*F*_(2, 18)_ = 3.41, *p* = 0.06], FMMA at week 0 [*F*_(2, 18)_ = 0.83, *p* = 0.45], and other demographic.

**Table 1:** **Demographics and baseline characteristics of subjects by intervention**.

		**Intervention**		
**Variable**	**Total**	**BCI-HK**	**HK**	**SAT**
N	21	6	8	7
Age (years)	54.2 ± 12.4	54.0 ± 8.9	51.1 ± 6.3	58.0 ± 19.3
**GENDER *N*(%)**				
Male	14 (66.7%)	4 (66.7%)	6 (75.0%)	4 (57.1%)
Female	7 (33.3%)	2 (33.3%)	2 (25.0%)	3 (42.9%)
**DOMINANT HAND AFFECTED *N*(%)**				
Yes	11 (52.4%)	2 (33.3%)	5 (62.5%)	4 (57.1%)
No	10 (47.6%)	4 (66.7%)	3 (37.5%)	3 (42.9%)
**STROKE TYPE *N*(%)**				
Infarction	11 (52.4%)	2 (33.3%)	4 (50.0%)	5 (71.4%)
Hemorrhage	10 (47.6%)	4 (66.7%)	4 (50.0%)	2 (28.6%)
**STROKE NATURE *N*(%)**				
Cortical	6 (28.6%)	1 (16.7%)	2 (25.0%)	3 (42.9%)
Subcortical	15 (71.4%)	5 (83.3%)	6 (75.0%)	4 (57.1%)
Duration since stroke (days)	385.1 ± 131.8	285.7 ± 64.0	398.2 ± 150.9	455.4 ± 109.6
FMMA (Week 0)	27.0 ± 13.8	33.0 ± 16.2	25.5 ± 11.5	23.4 ± 14.5

*FMMA, Fugl-Meyer Motor Assessment*.

### EEG spatial patterns and features

**Figure 5:**
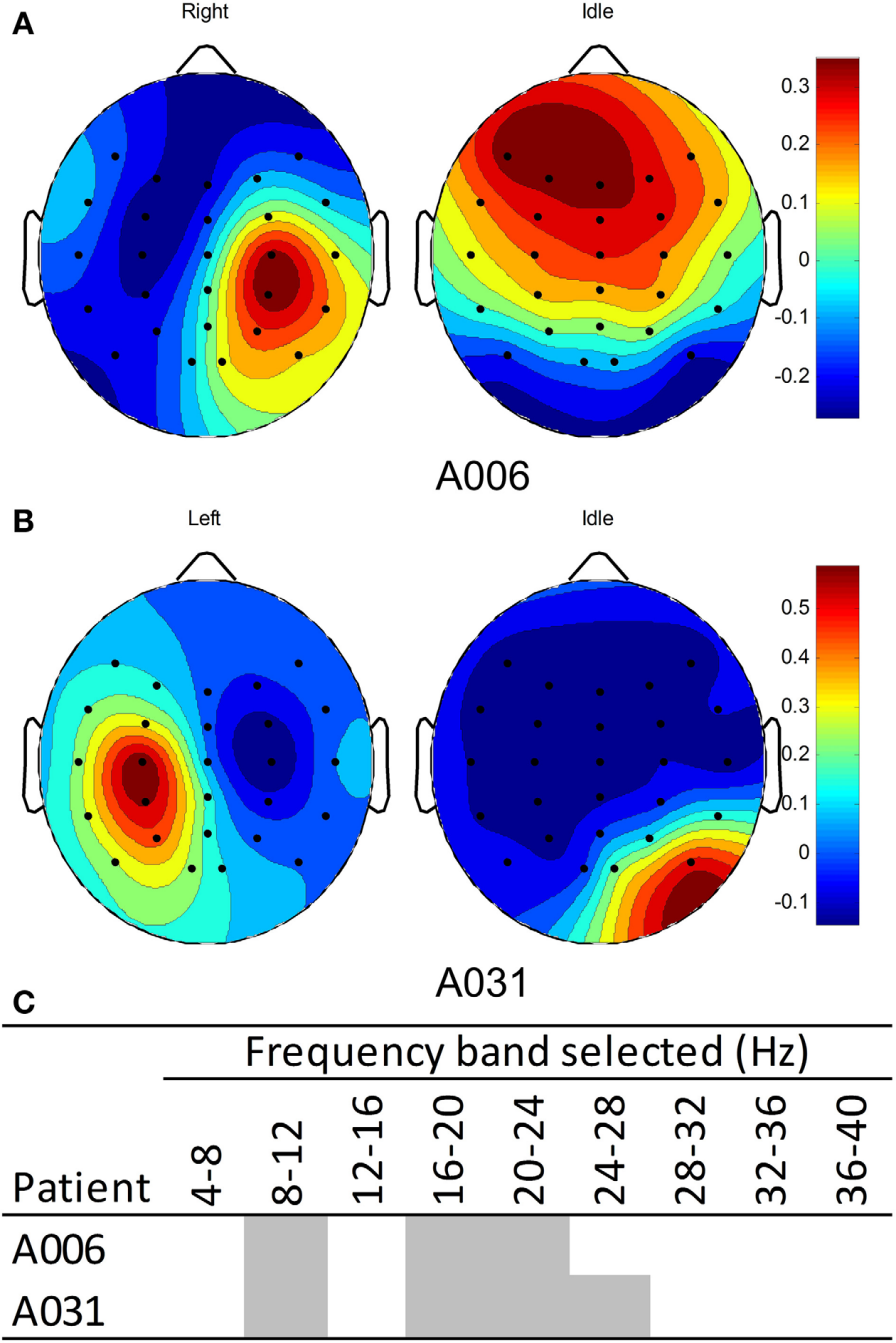
**EEG Spatial patterns and frequency bands used to classify motor imagery of stroke-impaired hand vs. idle condition. (A)** Spatial patterns of patient A006 with right stroke-impaired hand; **(B)** spatial pattern of patient A031 with left stroke-impaired hand; **(C)** frequency bands used for patients A006 and A031. Blue and red colors in the spatial patterns correspond to negative (ERD) and positive (ERS) values respectively.

The EEG from the calibration sessions of patients in the BCI-HK group were used to compute a subject-specific calibration model using the FBCSP algorithm (Ang et al., 2012). Figure 5A shows the EEG spatial patterns from patient A006 who performed MI of right stroke-impaired hand vs. the idle condition. The patterns for detecting MI-related brain signals of right hand showed a weak contra-lateral negative region on the left hemisphere and a relatively stronger ipsi-lateral positive region on the right hemisphere around the motor cortex area. The patterns from these two regions corresponded to ERD and ERS respectively for performing right hand motor imagery (Blankertz et al., 2008). Figure 5B shows the EEG spatial patterns from patient A031 who performed MI of left hand vs. the idle condition. Similarly, the patterns for detecting MI-related brain signals of left hand showed a weak contra-lateral negative region on the right hemisphere and a relatively stronger ipsi-lateral positive region on the left hemisphere around the motor cortex area. The patterns from these two regions corresponded to ERD and ERS respectively for performing left hand MI (Blankertz et al., 2008). The weaker stroke-affected contra-lateral regions compared to unaffected ipsi-lateral regions may be due to the relatively lower baseline ERD in stroke patients compared to healthy subjects reported in the study by Kasashima et al. (2012). For both patients, the EEG spatial patterns for the idle condition were not coherent since this condition was not controlled. Figure 5C shows the frequency bands selected by the FBCSP algorithm for both patients.

### Efficacy measurements

At week 6, upon completion of interventions, all groups demonstrated significant FMMA score gains compared to baseline FMMA score at week 0: BCI-HK group [(*M* = 7.2, *SD* = 2.3), *t*_(5)_ = 7.58, *p* = 0.001], HK group [(*M* = 7.3, *SD* = 4.7), *t*_(7)_ = 4.35, *p* = 0.003], and SAT group [(*M* = 4.9, *SD* = 4.1), *t*_(6)_ = 3.10, *p* = 0.021]. At weeks 12 and 24, significant FMMA score gains compared to baseline FMMA score at week 0 were sustained for BCI-HK group [(*M* = 8.2, *SD* = 2.9), *t*_(5)_ = 6.83, *p* = 0.001; and (*M* = 9.7, *SD* = 2.9), *t*_(5)_ = 8.04, *p* = 0.001] and HK group [(*M* = 6.5, *SD* = 4.4), *t*_(7)_ = 4.14, *p* = 0.004; and (*M* = 8.3, *SD* = 5.0), *t*_(7)_ = 4.66, *p* = 0.002]; but not for SAT group [(*M* = 3.6, *SD* = 5.5), *t*_(6)_ = 1.71, *p* = 0.14; and (*M* = 3.6, *SD* = 5.9), *t*_(6)_ = 1.60, *p* = 0.16] (Table 2).

**Table 2:** **Efficacy measures by FMMA scores for each intervention group (*N* = 6 for BCI-HK, *n* = 8 for HK, and *N* = 7 for SAT)**.

**Outcome**	**Group**	**Baseline**	**Improvements relative to week 0**			
		**Week 0**	**Week 3**	**Week 6**	**Week 12**	**Week 24**
Proximal (0~42)	BCI-HK	24.2 ± 7.5	3.3 ± 4.2	3.8 ± 2.7	5.0 ± 2.4	5.5 ± 2.1
	HK	19.5 ± 7.7	2.3 ± 2.7	4.4 ± 2.7	4.0 ± 3.5	5.8 ± 2.9
	SAT	18.1 ± 10.4	1.1 ± 2.2	3.0 ± 2.7	2.6 ± 4.4	3.3 ± 4.0
Distal (0~24)	BCI-HK	8.8 ± 9.2	2.5 ± 2.4	3.3 ± 2.3	3.2 ± 2.7	4.2 ± 3.1
	HK	6.0 ± 4.7	1.6 ± 2.5	2.9 ± 3.0	2.5 ± 2.6	2.5 ± 3.0
	SAT	5.3 ± 4.7	0.4 ± 1.1	1.9 ± 1.9	1.0 ± 1.3	0.3 ± 2.1
Upper Extremity (0~66)	BCI-HK	33.0 ± 16.2	5.8 ± 4.7	7.2 ± 2.3	8.2 ± 2.9	9.7 ± 2.9
	HK	25.5 ± 11.5	3.9 ± 4.3	7.3 ± 4.7	6.5 ± 4.4	8.3 ± 5.0
	SAT	23.4 ± 14.5	1.6 ± 2.2	4.9 ± 4.1	3.6 ± 5.5	3.6 ± 5.9

**Figure 6:**
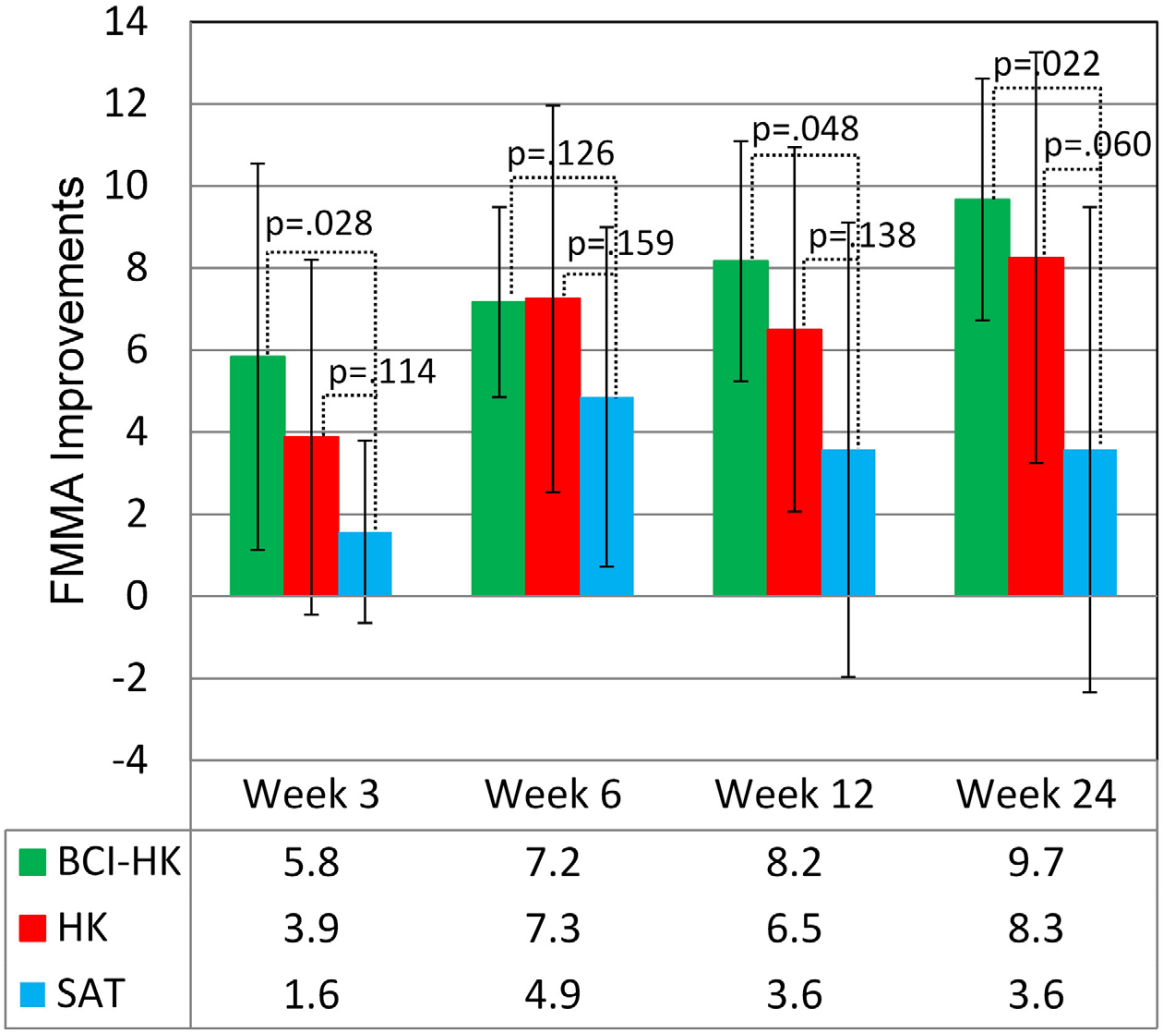
**FMMA improvements for BCI-HK, HK and SAT interventions relative to week 0**.

No significant intergroup differences were observed at any time point during the study among all the 3 groups after adjusting for baseline FMMA score at week 0: week 3 [*F*_(2, 17)_ = 1.51, *p* = 0.250], week 6 [*F*_(2, 17)_ = 0.66, *p* = 0.531], week 12 [*F*_(2, 17)_ = 1.12, *p* = 0.349], and week 24 [*F*_(2, 17)_ = 2.39, *p* = 0.122]. Significant greater upper extremity FMMA score gains were observed in the BCI-HK group compared to the SAT group at week 3 [*t*_(11)_ = 2.14, *p* = 0.028], week 12 [*t*_(11)_ = 1.82, *p* = 0.048], and week 24 [*t*_(11)_ = 2.28, *p* = 0.022]; but not at week 6, [*t*_(11)_ = 1.21, *p* = 0.13] (Figure 6). However, no significant greater FMMA score gains were observed in the HK group compared to the SAT group at any time point: week 3 [*t*_(13)_ = 1.27, *p* = 0.114], week 6 [*t*_(13)_ = 1.04, *p* = 0.159], week 12 [*t*_(13)_ = 1.14, *p* = 0.138], and week 24 [*t*_(13)_ = 1.66, *p* = 0.060] (Figure 6).

### Adverse events

There were no reported serious adverse events, deaths, or significant increases in shoulder or hand pain for any of the 3 intervention groups at any time during the study duration. One subject (4.6%) from the BCI-HK group dropped out in the 5^th^ week of intervention due to a transient mild seizure occurring several hours after the intervention.

## Discussion

This is the first RCT that compared 3 arms of MI-BCI, robot-assisted PP and SAT. Difficulties were encountered in recruiting patients for this study due to the strict inclusion and exclusion criteria. In addition, some patients who were clinically eligible did not pass the BCI screening or voluntarily declined to participate due to the length of the study.

The results on the discriminative spatial patterns and frequency band used to classify MI of stroke-impaired hand vs. the idle condition in the BCI-HK group differed from patient-to-patient, demonstrating the necessity to perform subject-specific calibration. The amount of movement repetitions were standardized between the BCI-HK and HK group. However, the number of arm repetitions were not measured in the SAT group, but the duration of training was similar with respect to the other 2 groups. The results showed significant efficacy in reducing both proximal and distal motor impairment, low dropout rate and safety. The results also showed the importance of distal training of the arm for proximal improvement, which is consistent with the study by Lambercy et al. (2011) on 15 chronic patients using the HK robot.

Compared to other chronic stroke patients in robot-assisted PP for proximal and distal (Lo et al., 2010; Lambercy et al., 2011), the FMMA score gains from the BCI-HK and HK groups were higher (~7 at week 6 vs. ~3 to 4). Possible reasons included a relatively younger stroke study cohort (mean age 54 years) and larger proportion of cerebral hemorrhages (~50%) compared to the Caucasian populations who typically have a higher proportion of infarctions.

FMMA score gains at week 6 for all 3 groups were sustained till week 24. Further gains of 2.5 and 1.0 were observed in the BCI-HK and HK groups, and a loss of 1.3 in the SAT group was observed at week 24 relative to week 6. This may be due to the reduction in motor impairment that facilitated further home-based PP.

A significant greater FMMA score gains were observed in the BCI-HK compared to the SAT group. This may be due to the performance of MI in the BCI-HK group that facilitated neuroplasticity, which was suggested from the functional Magnetic Resonance Imaging (fMRI) study on resting state changes in functional connectivity on patients who underwent BCI with robot-assisted rehabilitation after stroke by Varkuti et al. (2013). A greater FMMA score gains were also observed in the HK group compared to the SAT group, but the gains were not significant. This may be due to the highly repetitive and thus higher intensity of PP in the robot-assisted HK group compared to the therapist-assisted SAT group, but lacked the additional positive effects of MI in the BCI-HK group. Similar benefits of MI were seen in another study that investigated chronic stroke patients who received MI-BCI with hand and arm orthoses feedback vs. those who received random orthoses feedback not linked to BCI (Ramos-Murguialday et al., 2013), suggesting a possible role for BCI in rehabilitation for stroke.

### Study limitations

The major limitations of our study were its small sample size and under-powering. This was likely due to the strict criteria required for BCI-related training in terms of cognitive and attention requirements. Due to the small sample size, our results need to be interpreted with caution. Due to a younger and larger proporation of hemorrhagic strokes, which may be expected from a predominantly Chinese population (85.7%), results from our study may lack the ability for generalization as to how the general stroke population will respond to BCI-related rehabilitation. As the number of repetitions was not monitored for the SAT group, there was a lack of standardization on the number of PP trials for this group. In addition, the motor improvements measured by FMMA are limited by a ceiling effect and focused more on proximal arm (Gladstone et al., 2002), thus such gains may not directly translate to changes in activities of daily living.

## Conclusions

There was significant higher motor gain up to 6 months for subjects in the BCI-HK intervention compared with SAT. This adds support to the potential of BCI-HK coupled with rehabilitation therapy as an adjunctive rehabilitation tool for wrist and hand rehabilitation after chronic stroke. Overall side effects were minimal and interventions were well-tolerated. Additional research and larger studies are needed to study neuroplasticity-related changes from the use of BCI in stroke rehabilitation, and to enhance the portability and usability of BCI interfaces.

### Conflict of interest statement

The authors declare that the research was conducted in the absence of any commercial or financial relationships that could be construed as a potential conflict of interest.

## Footnotes

^1^Neuroscan Nuamps EEG Amplifier. Compumedics USA, Compumedics Neuroscan and Compumedics DWL, 6605 West W.T. Harris Blvd, Suite F, Charlotte, NC 28269, USA.
